# Evaluating the potential of the sterile insect technique for malaria control: relative fitness and mating compatibility between laboratory colonized and a wild population of *Anopheles arabiensis *from the Kruger National Park, South Africa

**DOI:** 10.1186/1756-3305-4-208

**Published:** 2011-10-31

**Authors:** Givemore Munhenga, Basil D Brooke, Tobias F Chirwa, Richard H Hunt, Maureen Coetzee, Danny Govender, Lizette L Koekemoer

**Affiliations:** 1 Vector Control Reference Unit, National Institute for Communicable Diseases of the National Health Laboratory Service, Private Bag X4, Sandringham, Johannesburg 2131, South Africa; 2 Malaria Entomology Research Unit, School of Pathology, Faculty of Health Sciences, University of the Witwatersrand, Johannesburg, South Africa; 3 Epidemiology and Biostatistics Division, School of Public Health, Faculty of Health Sciences, University of the Witwatersrand, Johannesburg, South Africa; 4 Scientific Services, South African National Parks, Private Bag X402, Skukuza, 1350, South Africa; 5 Department of Paraclinical Sciences, Faculty of Veterinary Science, University of Pretoria, Private Bag X04, Onderstepoort, 0110, South Africa

**Keywords:** Sterile Insect Technique, *Anopheles arabiensis*, malaria vector control

## Abstract

**Background:**

The successful suppression of a target insect population using the sterile insect technique (SIT) partly depends on the premise that the laboratory insects used for mass rearing are genetically compatible with the target population, that the mating competitiveness of laboratory reared males is at least comparable to that of their wild counterparts, and that mass rearing and sterilization processes do not in themselves compromise male fitness to a degree that precludes them from successfully competing for mates in the wild. This study investigated the fitness and sexual cross-compatibility between samples of field collected and laboratory reared *An. arabiensis *under laboratory conditions.

**Results:**

The physiological and reproductive fitness of the MALPAN laboratory strain is not substantially modified with respect to the field population at Malahlapanga. Further, a high degree of mating compatibility between MALPAN and the Malahlapanga population was established based on cross-mating experiments. Lastly, the morphological characteristics of hybrid ovarian polytene chromosomes further support the contention that the MALPAN laboratory colony and the *An. arabiensis *population at Malahlapanga are genetically homogenous and therefore compatible.

**Conclusions:**

It is concluded that the presence of a perennial and isolated population of *An. arabiensis *at Malahlapanga presents a unique opportunity for assessing the feasibility of SIT as a malaria vector control option. The MALPAN laboratory colony has retained sufficient enough measures of reproductive and physiological fitness to present as a suitable candidate for male sterilization, mass rearing and subsequent mass release of sterile males at Malahlapanga in order to further assess the feasibility of SIT in a field setting.

## Background

Following approval of a Ministerial Resolution by the Southern African Development Community (SADC) [1], South Africa is now listed as one of four countries in southern Africa (along with Botswana, Namibia and Swaziland) hoping to achieve malaria elimination status by 2015 [2].

Malaria vector control in South Africa is primarily based on indoor residual spraying (IRS) of insecticide. Although effective, this strategy has not completely halted malaria transmission in affected regions. This is partly due to the development of insecticide resistance in target populations of the major malaria vectors *Anopheles funestus *and *An. arabiensis *[3-5]. As a consequence of the development and spread of insecticide resistance as well as the South African National Department of Health (NDoH) thrust toward malaria elimination, additional strategies are needed to strengthen current vector control interventions.

The sterile insect technique (SIT) has been used widely and successfully to control a number of insect pest species. Examples include eradication of the New World screwworm fly, *Cochliomyia hominivorax*, from the USA, Mexico and all of Central America [6,7], control of the Mediterranean fruit fly, *Ceratitis capitata *[8], and eradication of the tsetse fly *Glossina austeni *from the Island of Zanzibar [9]. South Africa is currently successfully using SIT for the control of the Mediterranean fruit fly (Medfly) in the Hex River Valley [10]. Two additional agricultural insect pests are also being targeted using SIT, the codling moth and the false-codling moth [11].

The use of SIT to control mosquitoes dates back to the 1960s when sterile *Aedes aegypti *males were released in Florida, USA, with the aim of reducing *Aedes *populations in Pensacola [12]. This was followed by substantial research on mosquito SIT aimed at answering specific entomological questions rather than direct control [13]. Field trials done in the 1980s demonstrated that SIT could work against mosquitoes [13]. The experience and knowledge gained in the 1970s coupled with advances in transgenic technology has resulted in considerable interest in SIT for malaria vector control. There is currently a pilot field trial underway in northern Sudan to ascertain the feasibility of using SIT to control the African malaria vector *An. arabiensis *[14].

The sterile insect technique is based on the use of laboratory reared sterile males which are mass released into natural environments that differ from those in which they were reared. The aim is to suppress or eradicate target pest populations by disrupting the production of progeny. In order to achieve this, released sterile males should be able to compete against wild males for wild females under a wide range of environmental conditions. Sterile males used for SIT programmes are usually derived from insect colonies, which have been reared under laboratory conditions for several years. Colonization and mass-rearing of insects in this way can result in significant genetic divergence between colonized and wild populations [15,16]. Laboratory colonization can also lead to a significant loss in physiological and reproductive fitness as a consequence of reduced genetic variation induced by founder effects, bottlenecking and uniformity of a laboratory rearing environment [17].

The selection of deleterious phenotypes, such as abnormal mating behaviours, reduced fitness and sexual isolation, as consequences of colonization is an important consideration for the implementation of a SIT programme. For example, in insectary environments males do not necessarily search for and locate swarming markers. This may lead to confusion of laboratory reared males in terms of when or where to mate under natural conditions, reducing their measure of mating success in the wild [18]. Colonization has also been shown to promote consanguineous mating which results in reductions in fitness [19]. Colonization over time may also result in sexual isolation [15,16] leading to sexual incompatibility. Therefore, baseline information concerning the fitness and sexual compatibility between a laboratory colony ear-marked for mass releases and the targeted natural population must be investigated as a means of assessing the feasibility of SIT. This study compared mating success, fertility and fecundity between a long established laboratory reared colony and a wild population of *An. arabiensis *from the same geographical area in Malahlapanga, Kruger National Park, South Africa.

## Results

### Mosquito collections

In total, 559 anophelines belonging to four different taxa were collected during the 3 sampling periods. *Anopheles arabiensis *was the predominant species contributing 49.6% to the total collection, while *An. quadriannulatus*, another member of the *An. gambiae *complex, contributed 13.2% to the collection. The remaining 37.2% was composed of an assortment of anophelines including members of the *An. coustani *group, *An. maculipalpis *and *An. rivulorum *(Table 1). Human landing catches were not productive in the June 2010 collection as only six adults were caught. In the February 2011 collection members of the *An. coustani *group were predominant (52.8%).

**Table 1:** Anopheline mosquitoes caught at Malahlapanga, Kruger National Park, during three collection periods (HLC = Human Landing Catches; No = Sample size; - Identification to species-specific level was not done)

Collection period	Collection Method	**No**.	*An. arabiensis*	*An. quadriannulatus*	Other anophelines	*An. rivulorum*	*An. maculipalpis group*	*An. coustani group*
June, 2010	Larval HLC	114 6	18 (%) 4	22 (%) 1	74 (%) 1	-	-	-
November, 2010	HLC + CO_2_	295	223	23	49	-	-	-
February, 2011	HLC + CO_2_	144	32	28	84	(2)	(6)	(76)

**Totals (%)**		**559**	**277(49.6)**	**74(13.2)**	**208(37.2)**	**(2)**	**(6)**	**(76)**

### Measurement of fitness components

Fecundity of wild collected females and laboratory females could not be compared directly because wild-caught females were of unknown age. Therefore, their gonotrophic cycle, number of blood meals and blood source were not known. A total of 135 wild-caught *An. arabiensis *females were held for oviposition. Of these 67.7% (n = 85) produced at least one egg batch. The mean egg production for the wild collected females was 125 eggs/female with lower and upper 95% confidence intervals (CI) of 110.7 and 139.7 respectively. The minimum and maximum number of eggs laid was 8 and 425 respectively. The MALPAN females (n = 60; two replicates of 30 each) were held for egg laying seven days after mating and the provision of three blood meals. A mean egg production of 68.9 eggs/female and a lower and upper 95% CI of 50.4 and 81.7 respectively were recorded. The minimum and maximum egg production range for MALPAN females was 49 and 87 respectively.

**Figure 1:**
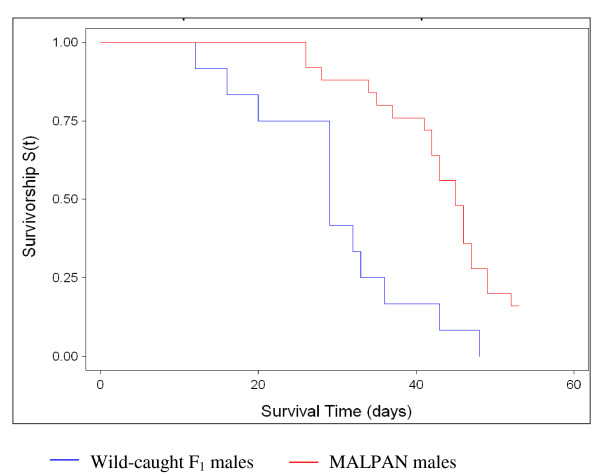
**Survivorship curves for MALPAN (laboratory) and wild-caught F_1 _males reared under standard insectary conditions**.

The mean duration and survival rates of the immature stages of the F_1 _progeny of wild-caught females and of MALPAN females are summarised in Table 2. There was no significant difference in egg hatch rates between the wild-caught F_1 _and MALPAN cohorts (Student's t-test, P = 0.44). However, egg batches from wild-caught females showed greater heterogeneity in hatching, varying between 28% and 100%, as opposed to that of MALPAN which varied from 56% to 93%. It took an average of five days for eggs from wild-caught females to hatch while the time-to-hatch period for MALPAN eggs was only three days. There was no significant difference in the proportion of larvae which survived and pupated between the two cohorts (Student's t-test, P = 0.22). The mean larval developmental time from first instar (L1) to pupal stage was significantly longer in MALPAN compared to the wild-caught sample (Student's t-test, P = 0.04). There was no significant difference in adult emergence rates (Student's t-test, P = 0.15) between the wild-caught samples and MALPAN. Analysis of adult emergence by gender showed that more females emerged in wild-caught samples (52.8%, n = 713) compared to 49.3% (n = 625) in MALPAN, but this difference is not significant (Student's t-test, P = 0.93). The transition time from pupae to adults showed no significant difference between the two cohorts (Student t-test, P = 0.78). Adult longevity assessments showed that MALPAN adults survived for significantly longer than the F_1 _progeny of wild-caught females (Table 3). In both cases males survived longer than females. Importantly, MALPAN males survived significantly longer than wild-caught F_1 _males (Cox's F test, F = 3.42, P = 0.002), (Figure 1).

**Table 2:** Mean duration and survival rates of immature stages of MALPAN (MALP) and Field collected F_1 _progeny.

Attributes	Egg Stage		Larval Stage L1-L4*		Pupal Stage	
	
	**Field F**_**1**_	MALP	**Field F**_**1**_	MALP	**Field F**_**1**_	MALP
**Egg-hatching rate (%) ± SD (95% CI)**	78.8 ± 25.7 (73.2 - 84.5)	68.7 ± 11.2 (65.3 - 72.1)	-	-	-	-
**Duration of Immature stage ± SD (95% CI)**	4.7 ± 0.3 (4.2 - 5.1	3.1 ± 0.6 (2.7 - 3.6)	7.3 ± 1.2 (6.6 - 7.9)	9.24 ± 1.6† (8.5 - 9.9)	2.5 ± 0.6 (2.1 - 2.8)	2 ± 0.8 (1.9 - 2.3)
**Survival rate ± SD** **(95% CI)**	-	-	85.4 ± 16.4 (77.9 - 92.9)	86.5 ± 10.0 (70.6 - 92.3)	96.4 ± 6.2 (93.5 -99.3)	90.3 ± 6.9 (88.7 - 95.4)

* L1- L4 first, second, third and fourth larval instar, † showed statistically significant difference

**Table 3:** Mean survival times of adult *Anopheles arabiensis *laboratory reared (MALPAN) and the F_1 _progeny of wild-caught females reared under standard insectary conditions

	**Field F**_**1**_		Malpan	
	
	Males	Females	Males	Females
**Mean survival time (days)**	29	26*^†^	45^†^	31*
**Lower - Upper 95% CI**	20-36	19-29	42-47	28-32

* Statistically significant difference between males and females within strain

† Statistically significant difference between males and females between strains

### Mating compatibility studies

**Figure 2:**
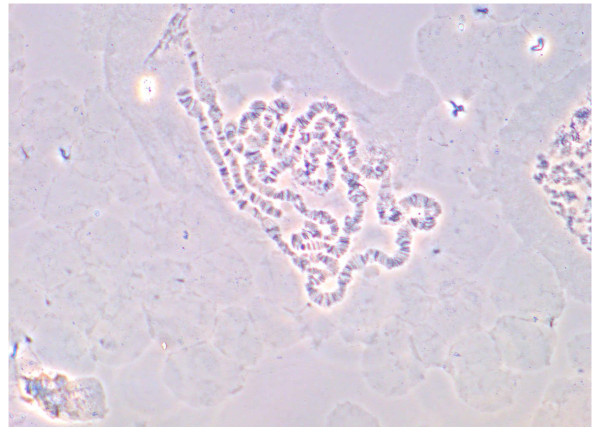
**Hybrid ovarian polytene chromosomes from a hybrid female produced by crossing wild-caught F_1 _and MALPAN *Anopheles arabiensis *samples**.

Mating between the F_1 _progeny of wild-caught samples and MALPAN was successful. Table 4 summarises mean wing lengths, insemination rates and egg production of the four crosses. The highest insemination rate was recorded in females from the cross involving MALPAN males and F_1 _females. Females from the cross between F_1 _males and F_1 _females showed the lowest rate of insemination. Variation in insemination rates between the four cross permutations was significant (ANOVA, DF = 3, P = 0.02). Females from each cross mating experiment produced eggs. The highest mean egg production was recorded from the cross between MALPAN females and F_1 _males whilst the lowest was recorded in the cross between MALPAN females and MALPAN males. Variation in mean egg production per female between the four cross permutations was significant (ANOVA, DF = 3; P = 0.02). Comparisons of means using Bonferroni comparison showed that there was a significant difference in mean egg production per female between F_1 _females crossed with MALPAN males and F_1 _males crossed with MALPAN females (Student's t-test, P = 0.047). The mean duration and survival rates of immature stages of progeny from the crosses are tabulated on Table 5. The mean embryony development (time-to-hatch) of oviposited eggs showed a significant difference between the crosses (ANOVA, DF = 3, P = 0.001). Bonferroni comparison of means showed that eggs oviposited from the cross between MALPAN males and MALPAN females required a significantly shorter time (3 days) to hatch compared to the other cross permutations. There was significant variation in the mean proportions of larvae pupating between the four cross permutations (ANOVA, DF = 3, P = 0.04). Proportional adult emergence rates showed no significant difference between the cross permutations (ANOVA, DF = 3, P = 0.56). Emergence by gender showed that F_1 _females produced more female progeny than MALPAN females regardless of whether they were crossed with F_1 _or MALPAN males (Table 5). Hybrid ovarian polytene chromosomes dissected from hybrid female progeny produced by crossing wild-caught F_1 _and MALPAN samples appeared normal with no obvious signs of asynapsis (Figure 2).

**Table 4:** Mean wing length, % insemination rates and mean egg production/female of F_1 _progeny females from crosses between wild-caught F_1_'s and MALPAN *An. arabiensis *(F = Field, Mal = MALPAN, ♀ = female, ♂ = male, × = crossed with)

	Cross			
	
Attribute	F♀ X F♂	F♀ X Mal♂	Mal♀ X F♂	Mal♀ X Mal♂
**Mean wing length (mm)** **(Lower - Upper 95% CI)**	3.69 3.59 - 3.8	3.61 3.48 - 3.75	3.64 3.52 - 3.76	3.66 3.56 - 3.76
**% Insemination rate** **(Lower - Upper 95% CI)**	37.9 35.4 - 40.3	72.7† (66.4 - 79.7)	49.3 (45.7 - 52.9)	53.8 (50.8 - 56.8)
**Mean egg production/female** **(Lower - Upper 95% CI)**	76.2 (50.4 - 102)	62 (47.3 - 72.7)	141.3† (134.4 - 155.1)	58.7 (29.7 - 87.6)

† showed statistically significant difference

**Table 5:** Mean duration and survival rates of the immature stages of progeny from crosses between wild-caught F_1_'s and MALPAN *An. arabiensis *(F = Field, Mal = MALPAN, ♀ = female, ♂ = male, × = crossed with)

Attributes	Egg Stage				Larval Stage L1-L4				Pupal Stage			
	
	F♀ X F♂	F♀ X Mal♂	Mal♀ X F♂	Mal♀ X Mal♂	F♀ X F♂	F♀ X Mal♂	Mal♀ X F♂	Mal♀ X Mal♂	F♀ X F♂	F♀ X Mal♂	Mal♀ X F♂	Mal♀ X Mal♂
**Egg-hatching rate (%)± SD**	72.5 ± 25.8	57.9 ± 28.2	76.0 ± 21.1	82.6 ± 13.7	-	-	-	-	-	-	-	-
**Survival rate **(%) **± SD**	-	-	-	-	94.9 ± 4.9	95.6 ± 3.9	93.5 ± 6.0	86.2† ± 12.5	93.9 ± 5.8	96.7 ± 2.1	91.0 ± 7.5	93.9 ± 8.1
**Duration of Immature stage ± SD**	4.5 ± 0.4	4.4 ± 0.5	3.5 ± 0.9	2.9† ± 0.7	-	-	-	-	-	-	-	
**% of females emerging**	-	-	-	-	-	-	-	-	50.8	52.9	46.4	43.9

† showed statistically significant difference

## Discussion

The continued presence of a perennial population of *An. arabiensis *at Malahlapanga was confirmed based on the seasonal collections described. The collection data also showed a significant seasonal trend in relative abundance compared to other anopheline species that also occurred there, whereby *An. arabiensis *were least abundant in the dry winter months and most abundant following the onset of rains in early summer. This seasonal fluctuation is an important consideration for SIT, because mass release of sterile males is most effective when the target population abundance is low and the ratio between sterile and wild males is maximized [8].

The successful suppression of a target insect population using the sterile insect technique also depends on the premise that the laboratory insects used for mass rearing are genetically compatible with the target population, that the mating competitiveness of laboratory reared males is at least comparable to that of their wild counterparts, and that the mass rearing and sterilization processes do not in themselves compromise male fitness to a degree that precludes them from successfully competing for mates in the wild. Our data showed that the physiological and reproductive fitness of the *An. arabiensis *MALPAN laboratory strain is not substantially modified with respect to the field population at Malahlapanga. This is especially true of parameters including male longevity whereby MALPAN males showed greater longevity than the F_1 _male progeny of wild-caught females from Malahlapanga. In general, measures of fecundity, fertility and life stage duration compared favourably between MALPAN and the Malahlapanga samples. The reduced variation apparent in the fitness assessment parameters for MALPAN is likely a consequence of the reduced genetic variation generally inherent in laboratory colonies as compared to the wild populations from which they are derived.

Also of importance are the data accruing from the crosses between the MALPAN colony and the F_1 _progeny of wild-caught Malahlapanga females where F_1 _females showed the highest rate of insemination when crossed with MALPAN males, indicating a high degree of mating compatibility between the MALPAN laboratory colony and the Malahlapanga population. Comparative fitness parameters of the progeny produced from each of the cross-mating experiments support a high degree of genetic compatibility between MALPAN and the Malahlapanga population. Measures of fertility, fecundity and life stage duration of progeny were generally comparable between the MALPAN/Malahlapanga intercrosses and the sample intracrosses (MALPAN/MALPAN and Malahlapanga/Malahlapanga). Lastly, the complete absence of asynapsis in the hybrid ovarian polytene chromosomes further supports the contention that the MALPAN laboratory colony and the *An. arabiensis *population at Malahlapanga are genetically homogenous and therefore compatible. Hassan et al. [20] conducted compatibility experiments between colonized *An. arabiensis *and wild populations from Sudan, showing that the colonized strain was compatible with and mated with wild females.

## Conclusions

The presence of a perennial and geographically isolated population of *An. arabiensis *at Malahlapanga presents a unique opportunity for assessing the feasibility of SIT as a malaria vector control option. The MALPAN laboratory colony, which is derived from material collected at Malahlapanga, has for over 20 years retained sufficient reproductive and physiological fitness to present as a suitable candidate for male sterilization, mass rearing and subsequent mass release of sterile males at Malahlapanga in order to further assess the feasibility of SIT in a field setting.

## Materials and methods

### Study site

**Figure 3:**
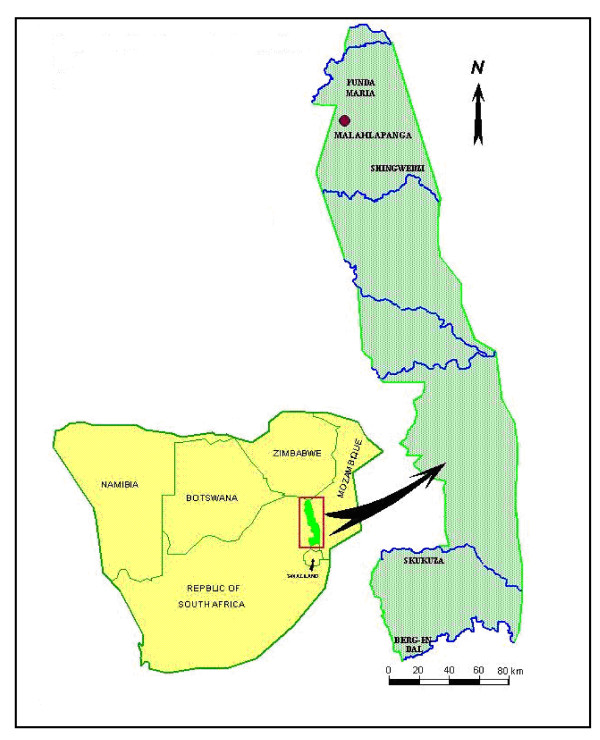
**Map of the Kruger National Park, South Africa, showing location of the sampling site at Malahlapanga (Modified from http://www.tropmed.org/rreh/vol1_12_files/image003.jpghttp://www.places.co.za/html/8009.html**.

Malahlapanga, (22°53'S; 31°02'E) is a fresh water geothermal spring situated in the Northwestern region of the Kruger National Park (Figure 3). The spring is surrounded by *Colophospermum mopane *and *Acacia nigrescens *trees [21]. Warm water (~37°C) from the eye of the spring flows downstream, creating a wide wetland with a profusion of suitable breeding sites for mosquitoes. The spring supports a perennial, geographically isolated, population of *An. arabiensis *[21]. Proliferation of mosquitoes is supported by abundant ruminant and antelope herds which use the pan as a water source.

### Anopheles arabiensis laboratory colony

The *An. arabiensis *laboratory colony, MALPAN, originates from material collected at Malahlapanga in 1994. It is maintained in the Botha DeMeillon insectary, Vector Control Reference Unit (VCRU), National Institute for Communicable Diseases (NICD), Johannesburg, South Africa, under standard insectary conditions of 25°C, 85% relative humidity and a photo period of 12: 12 hour light/darkness, with a 45-min dawn and dusk light regime.

### Wild mosquito collections

Mosquitoes were collected in June 2010, November 2010 and February 2011 from Malahlapanga. Host seeking females were collected outdoors by human landing catches and CO_2 _bait net traps between 18h00 and 23h00 over three successive nights in each collection month. Only six adults were collected in June 2010 as a consequence of adverse weather conditions, so larvae were collected instead. Larval sampling was based on the standard dipping method [22]. Only anopheline larvae were retained and preserved in 70% ethanol for further identification to species level in the laboratory. Adult mosquitoes were morphologically identified using a taxonomic key [23]. Those identified as members of the *An. gambiae *complex were pooled and maintained live in 250 ml paper cups for transport to the VCRU laboratory in Johannesburg. All wild-caught females were offered two blood meals in order to procure progeny for the series of experiments described below.

### Species identification

Specimens morphologically identified as members of the *An. gambiae *complex were identified to species level using the polymerase chain reaction (PCR) assay [24]. To get an overview of anopheline species composition in Malahlapanga other anophelines collected in February 2011 were identified to species level. Specimens identified as members of the *An. funestus *group were processed using the multiplex PCR assay with slight modifications [25].

### Measurement of fitness components

Comparative fitness between wild *An. arabiensis *from Malahlapanga and the MALPAN laboratory colony was assessed using the following fitness parameters:

#### (i) Fecundity

Individual *An. arabiensis *females collected from Malahlapanga were transferred into oviposition vials following blood feeding. Each oviposition vial contained a moistened Whatman filter paper disc (Cat No. 1001125) to induce oviposition. A total of 70 newly emerged males and 50 females from the MALPAN colony were allowed to mate for 7 days in 5-litre plastic adult cages before being offered three blood meals over a five day period. Immediately after the third blood meal, 30 randomly selected blood-fed females were placed in oviposition glass vials to induce oviposition. Oviposited eggs from each female were counted using a hand-held magnifying lens. Mean number of eggs laid were calculated. Fecundity of wild collected females and laboratory females could not be compared directly because wild-caught females were of unknown age. Owing to variation in the number of egg batches produced by each female, fecundity was scored as the number of eggs laid by each female per gonotrophic cycle.

#### (ii) Egg hatch rates

For each wild-caught egg batch and MALPAN female, eggs from the first gonotrophic cycle were transferred into plastic bowls (27 cm × 16 cm × 6.5 cm) containing 150 ml of distilled water and allowed to hatch. Upon hatching, larvae were counted and transferred to a new bowl of water. This was done daily for 10 consecutive days. The mean number of days taken to hatch and proportion of eggs hatching was determined and compared between the wild-caught and MALPAN samples.

#### (iii) Larval survivorship

Fifty randomly selected newly hatched larvae from eggs oviposited by each female cohort were placed in larval bowls (34 × 27 cm) containing 150 ml of distilled water. The larvae were fed daily on approximately 30 mg of larval food ((a mixture of brewers yeast (Vital Health Foods, South Africa) and finely ground dog biscuits (West's traditional crunching biscuits treats, Martin and Martin, South Africa)) prepared at a ratio of 1:3. Larvae were maintained under standard insectary conditions. Each day the numbers of surviving larvae were counted and their stage of development was recorded. Pupae that emerged were counted and transferred into pupal emergence vials (35 mm × 57 mm) placed in 10-litre adult emergence cages. Larval survivorship was calculated as the proportion of first-instar larvae which pupated. Mean larval survivorship was compared between the wild-caught and MALPAN samples.

#### (iv) Adult emergence and sex ratio

Pupae were counted by family and transferred into plastic vials filled with 50 ml distilled water and kept in 10-litre plastic cages for adult emergence. Pupae were monitored daily and the number and sex of emerging adults was recorded for each family. Only adult mosquitoes that successfully emerged and were capable of flying were scored as emerged. The mean proportion of pupae surviving to the adult stage was calculated for each strain and compared between the wild-caught and MALPAN samples.

#### (v) Adult longevity

Newly emerged F_1 _progeny from field collected females were pooled from at least 10 families, separated by sex and placed in mosquito cages (50 for each sex). For the laboratory colony (MALPAN), one-day old males and females (50 of each) were set up in cages as described above. All adults were then maintained on 10% sugar solution soaked on cotton wool at standard insectary conditions. Survival was assessed daily until 100% mortality was reached. The experiment was replicated three times.

#### (vi) Wing length measurements

Wing length gives a good approximation of body size [26]. Wing lengths were measured from all females used for fecundity studies as well as 50 males each from MALPAN and the F_1 _progeny of wild caught females. One wing was dissected from each individual and placed on a clean glass slide. Wing-lengths (wing tip to thorax joint) were measured under a calibrated dissecting microscope. Mean wing-length was compared between the wild-caught and MALPAN samples by gender.

### Mating compatibility

Mating compatibility between the F_1 _progeny of wild-caught samples and MALPAN was determined by combining fifty newly emerged females and 70 newly emerged males in 5 litre cages using the following combinations (i) MALPAN females × F_1 _males, (ii) MALPAN males × F_1 _females, (iii) F_1 _females × F_1 _males and (iv) MALPAN males × MALPAN females. Each cross was set up in duplicate. Mosquitoes from each cross were allowed to mate for seven days post-emergence. During this period they were maintained on a 10% sucrose solution under standard insectary conditions. After seven days the females were offered three successive blood meals with one day in between each blood meal. The following parameters were used to assess mating compatibility and fitness: insemination rate, fecundity, egg hatch rates, larval survivorship, pupal survivorship, emergence rates and adult survivorship of progeny from each cross.

#### (i) Insemination rates

After the third blood meal a sub-sample of 10 females from each cross described above was removed in order to determine the proportion/rate of insemination. Each female's spermatheca was dissected and the presence of spermatozoa was assessed under a dissecting microscope (Wild, Heerbrugg M5-71661, Switzerland) at 200 × magnification. The proportion of inseminated females was calculated for each cross enabling direct comparisons between cross-mating permutations.

Fecundity, egg hatch rates, larval survivorship, pupal survivorship, emergence rates and adult survivorship for each cross were determined as previously described above and a comparison between the crosses was carried out.

#### (ii) Hybrid polytene chromosome assessments

Seventy newly emerged F_1 _males from wild caught females and fifty one day old virgin females from MALPAN were pooled into a single 5 litre plastic cage and allowed to mate for seven days. Hybrid progeny accruing from this cross were raised to adults, placed in a cage and allowed to mate for seven days. After the seventh day females were offered three successive blood meals over a period of a week. After the third blood meal a petri dish painted black on the outside and filled with 40 mls of distilled water was put into the cage to induce oviposition. Two days after oviposition 10 hybrid females which had completed their gonotrophic cycle were randomly selected, transferred into a 250 ml paper cup and were offered another blood meal. Their ovaries were dissected at the half gravid stage and were then fixed in Carnoy's fixative (3 parts absolute alcohol: 1 part glacial acetic acid) for at least three days. Polytene chromosomes were then prepared for analysis [27,28]. The hybrid chromosomal preparations were examined under a phase contrast compound microscope (OLYMPUS BX50) in order to assess chromosomal homogeneity and to monitor abnormalities in the hybrid chromosomes such as asynapsis that could indicate a genetic discontinuity between the parental groups.

### Statistical analysis

Data on fecundity, adult sizes (wing length), hatch rates, larval survivorship and adult emergence was summarised as mean wing lengths, mean number of eggs produced, mean proportion of eggs hatching, mean proportion of larvae surviving to pupae and mean proportion of pupae surviving to adult stages respectively. A student-t test in Statistix 7^®^was used to analyze differences in mean egg production and adult sizes between wild-caught F_1 _progeny and MALPAN females. One way ANOVA was used to assess the differences in mean egg production between the crosses followed by Bonferroni comparison of means. Percentage values for larval and pupal survivorship as well as adult emergence and insemination rates of individual females were checked for normality and transformed where applicable to achieve normal distribution. These were then compared between wild-caught F_1 _progeny and MALPAN females using Student-t tests whilst for the crosses ANOVA was used to compare differences. Survival curves were analysed using Kaplan Meier survival analysis in XLSTAT^® ^2009 and Cox's F test was used to compare mean difference in survivorship between male and female cohorts as well as between samples. In all cases a P-value of less than 0.05 was considered to indicate statistical significance.

## Competing interests

The authors declare that they have no competing interests.

## Authors' contributions

GM carried out field work, species-specific identification, life table analysis, interpretation of results and wrote the first and subsequent drafts of the manuscript.

BDB was involved in field work, experimental design, interpretation of results and contributed to the writing of the manuscript. TFC helped in statistical analysis and contributed to the writing of the manuscript. RHH was involved in field work, morphological identification of anophelines and provided comments on the manuscript. MC was involved in the implementation of the project and contributed to the writing of the manuscript. DG helped with field logististics, provided comments on the manuscript. LLK conceived the project, oversaw its implementation, assisted with fieldwork, species identification and contributed to the subsequent writing of the manuscript. All authors read and approved the final version of the manuscript
